# Supplementary material for: Deworming coverage and its determinants among 12–59 months old children in East Africa: A population-based study
Source: PLoS One. 2024 Feb 1;19(2):e0297377. doi: 10.1371/journal.pone.0297377 (PMC10833511; doi:10.1371/journal.pone.0297377)
Supplement: Attachment — Submitted filename: Review comments on Manuscript Number.doc [file pone.0297377.s001.doc]

**Review comments on Manuscript Number: PONE-D-23-32930 “Deworming coverage and determinates among 12-59 months old children in East Africa: a population-based study using recent East African national survey data of 2011-2022"**

**Overall, the idea of research is very interesting to be studied nowadays** and paper is coherently developed. However, there are some comments and suggestions.

General comments the authors need to review the manuscript grammar and editing.

**Title**

- You may consider change the title to [Deworming coverage and determinates among 12-59 months old children in East Africa: a population-based study]

**Abstract**

- Change the subtitle [introduction] to [background and objectives] or [background and aim]. And try to make it more concise.
- It is recommended to write keywords in alphabetical order.
- You may consider changing the keywords to more reprehensive words [Deworming, Soil-transmitted helminth infections, tropical diseases…]
- The results section is relatively long with extensive statistical results that are clear enough in the results and tables. Try to make it more concise.

**Introduction**

- Well structured

**Subjects and methods**

- The inclusion/exclusion criteria are not clear. Are there any factors may affect the results.

**Statistical analysis**

- Well structured

**Discussion**

- Well structured
